# Compensatory T-Cell Regulation in Unaffected Relatives of SLE Patients, and Opposite IL-2/CD25-Mediated Effects Suggested by Coreferentiality Modeling

**DOI:** 10.1371/journal.pone.0033992

**Published:** 2012-03-29

**Authors:** Constantin Fesel, Marta Barreto, Ricardo C. Ferreira, Nuno Costa, Lara L. Venda, Clara Pereira, Claudia Carvalho, Maria Francisca Morães-Fontes, Carlos M. Ferreira, Carlos Vasconcelos, João F. Viana, Eugenia Santos, Berta Martins, Jocelyne Demengeot, Astrid M. Vicente

**Affiliations:** 1 Instituto Gulbenkian de Ciência, Oeiras, Portugal; 2 Associação dos Doentes com Lupus, Lisboa, Portugal; 3 Centro Hospitalar Lisboa Ocidental, EPE, Lisboa, Portugal; 4 Instituto de Ciências Biomédicas Abel Salazar, Porto, Portugal; 5 Instituto Nacional de Saúde Dr Ricardo Jorge, IP, Lisboa, Portugal; 6 Juvenile Diabetes Research Foundation/Wellcome Trust Diabetes and Inflammation Laboratory, Department of Medical Genetics, Cambridge Institute for Medical Research, University of Cambridge, Cambridge, United Kingdom; 7 Department of Physiology, Anatomy and Genetics, University of Oxford, Oxford, United Kingdom; 8 Clínica Universitária de Medicina II, Hospital de Santa Maria, Lisboa, Portugal; 9 Unidade de Imunologia Clínica, Hospital de Santo António, EPE, Porto, Portugal; University of Leuven, Rega Institute, Belgium

## Abstract

In human systemic lupus erythematosus (SLE), diverse autoantibodies accumulate over years before disease manifestation. Unaffected relatives of SLE patients frequently share a sustained production of autoantibodies with indiscriminable specificity, usually without ever acquiring the disease. We studied relations of IgG autoantibody profiles and peripheral blood activated regulatory T-cells (aTregs), represented by CD4^+^CD25^bright^ T-cells that were regularly 70–90% Foxp3^+^. We found consistent positive correlations of broad-range as well as specific SLE-associated IgG with aTreg frequencies within unaffected relatives, but not patients or unrelated controls. Our interpretation: unaffected relatives with shared genetic factors compensated pathogenic effects by aTregs engaged in parallel with the individual autoantibody production. To study this further, we applied a novel analytic approach named coreferentiality that tests the indirect relatedness of parameters in respect to multivariate phenotype data. Results show that independently of their direct correlation, aTreg frequencies and specific SLE-associated IgG were likely functionally related in unaffected relatives: they significantly parallelled each other in their relations to broad-range immunoblot autoantibody profiles. In unaffected relatives, we also found coreferential effects of genetic variation in the loci encoding IL-2 and CD25. A model of CD25 functional genetic effects constructed by coreferentiality maximization suggests that IL-2-CD25 interaction, likely stimulating aTregs in unaffected relatives, had an opposed effect in SLE patients, presumably triggering primarily T-effector cells in this group. Coreferentiality modeling as we do it here could also be useful in other contexts, particularly to explore combined functional genetic effects.

## Introduction

IgG autoantibodies are the diagnostic hallmark of Systemic Lupus Erythematosus (SLE). They show particularly high affinity for nucleic acid (dsDNA) as well as for nuclear and cytoplasmic proteins that physically interact with nucleic acids, although individual reactivity patterns are highly heterogeneous. Autoantibody generation, in part originating from inappropriately processed self-antigens [1], is clearly T cell-dependent and occurs during germinal center reactions leading to a high degree of somatic hypermutation [2]. In these reactions, T- and B-cell targets can diversify by intra- and intermolecular epitope spreading, a mechanism in which crossreactivity and aberrant interactions between specific T- and B-cells favor the selection of self-reactive B-cells. Epitope spreading in SLE appears primarily triggered by reactivity to Ro60/SSA and Sm/nRNP autoantigens [3]–[6]. SLE-type autoantibodies accumulate and diversify over many years before SLE is diagnosed [7]. These autoantibodies, however, do not necessarily indicate manifest disease, and self-reactive IgG with specificities indistinguishable from SLE patients is frequently found in their healthy relatives [8]–[10]. These unaffected relatives obviously share a genetic disposition to produce SLE-associated autoantibodies [10], although they usually remain disease-free for life.

Effector T-cells undergo regulation by Foxp3^+^ regulatory T-cells (Treg), which have raised extraordinary interest since it was demonstrated that their deficiency, depletion or alteration results in lymphoproliferative and autoimmune disorders in many contexts. Activated CD4^+^Foxp3^+^CD25^bright^ Tregs (aTreg), recently characterized in humans [11], express high levels of CD25, the specific α subunit that defines the high-affinity IL-2 receptor. It is well established that IL-2 produced by activated CD4^+^ effector T-cells is essential for maintenance and proliferation of Foxp3^+^CD25^bright^ Tregs [12]. Consequently, mice deficient for IL-2 [13], [14], CD25 [15], or the IL-2 receptor β subunit [16], developed uncontrolled lymphoproliferation and lethal autoimmunity. In human SLE, a relative deficiency of IL-2 production in activated T-cells, by a mechanism of transcriptional repression, is well documented [17], and a functional relevance of impaired IL-2 production was directly demonstrated for lupus manifestation in NZB/W F1 mice [18], as it had been for other mouse autoimmunity models [19]. Accordingly, indications of Treg abnormalities in human SLE, such as reduced frequencies of aTregs [20]–[23] as well as reversible impairment of their functionality [24] have repeatedly been reported. Particularly, a characteristically increased population of CD4^+^Foxp3^+^ cells with low or absent surface CD25 [25], [26] and reduced suppresive capacity, but maintaining other Treg properties [27], [28] has pointed to an altered role of IL-2. Treg frequencies measured on the basis of high surface CD25, thus representing functionally active Tregs, were also found negatively correlated with anti-dsDNA antibodies [23] and disease activity [22]. We have previously reported the heritability of Treg frequencies in SLE-affected families [29]. However, unaffected relatives of SLE patients with SLE-type autoantibody reactivity have never been studied for properties of T-cell regulation. This is the subject of the present study: to investigate Tregs and their relationship with autoantibodies and IL-2 in unaffected relatives compared to the SLE patients themselves as well as to unrelated control subjects.

Given the uncharacterized heterogeneity of autoantibody patterns in unaffected relatives [8], in order to interpret their collective properties, a systematic broad-ranged study appeared appropriate. An adequate method for this, which will be used here in addition to specific IgG measurements, is the parallel and quantitatively standardized assessment of a multitude of autoreactive IgG specificities by quantitative immunoblot [30], as it has previously been applied to characterize antibody profiles in different contexts in animal [31]–[34] and human [35]–[39] studies. Furthermore, human genetic polymorphisms are deeply informative and regularly allow to identify risk factors for disease, but their use to study specific molecular or cellular mechanisms is very limited. A new analytical method, developed by us and named coreferentiality [40], will be used here to identify and characterize IL-2/CD25-mediated effects on broad-scale autoantibody profiles, through the analysis of genotypes in the loci encoding IL-2 and CD25.

We report the novel finding that various IgG autoantibody measures were consistently positively correlated with aTreg frequencies in unaffected relatives. We interpret these correlations as the result of a mechanism of efficient compensatory T-cell regulation, with a likely role of IL-2, that breaks down in manifest SLE. Coreferentiality analysis, allowing to test the functional relatedness of two parameters not in terms of direct correlation but in respect to multivariate reference data, was used to indirectly examine IL-2-mediated effects in this context through an analysis of genetic variation in the *IL2* and *IL2RA* (CD25) loci. In our unaffected relatives, we detected significant coreferentiality between genetic variants in these two loci in respect to immunoblot autoantibody profiles. Subsequently modeling the functional effect of *IL2RA* genetic variation and relating it to aTreg frequencies, it turned out that IL-2-CD25 interaction was indeed likely to trigger aTregs in the unaffected relatives, while in SLE patients IL-2/CD25-mediated effects were rather opposed to aTreg effects, supporting the notion that IL-2 triggered primarily T-effector cells in manifest SLE. More generally, we argue that the coreferentiality method has the power to model combined functional genetic effects, which may be very useful in many respects.

## Results

### 1. Multispecific and SLE-associated IgG autoantibody reactivity in unaffected relatives is intermediate between SLE patients and unrelated control subjects

**Figure 1:**
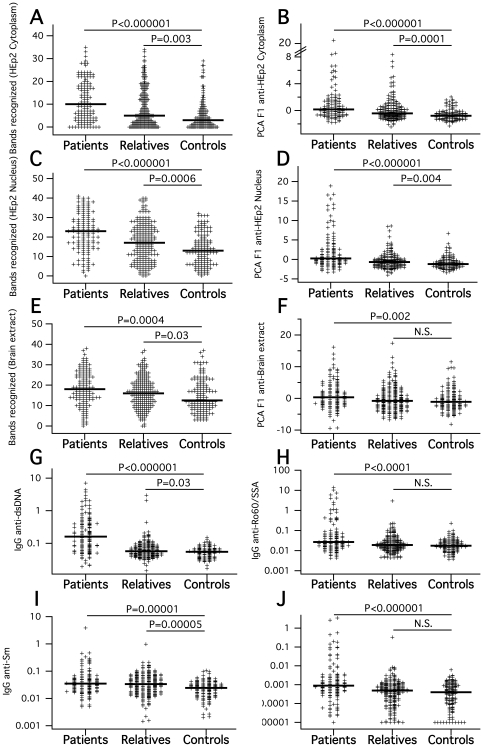
IgG autoreactivities in SLE patients, unafffected relatives and unrelated control subjects. A–F: Band numbers and 1st principal component calculated from HEp2 anti–cytoplasmic (A,B), anti-nuclear (C,D) and anti-brain (E,F) imunoblot reactivities. G–J: Specific SLE-associated autoreactive IgG quantified by ELISA. All plots show group-wise medians and results of pairwise Mann-Whitney tests for differences between groups.

We assessed quantitatively standardized immunoblot profiles of plasma IgG autoantibody reactivity from 128 SLE patients, 215 unaffected relatives and 140 healthy control subjects (listed in Table 1) to electrophoretically separated protein extracts of nuclear and cytoplasmic fractions of HEp2 cells as well as of human brain proteins. In these three immunoblot assays, performed in parallel for all subjects studied, plasma samples were diluted so that they had identical total protein concentrations. Reactivity patterns revealed on one of a total of 72 membranes are shown in Fig. S1. We could distinguish 46 separate reactivity bands to HEp2 cytoplasmic proteins, 38 to HEp2 nuclear proteins and 46 to brain proteins, adding up to a total of 130. They were densitometrically quantified and standardized (see methods). For each of the three extracts, we determined the band number recognized by IgG in each plasma sample. While SLE patients always recognized the highest median number of bands, unaffected relatives also recognized a significantly higher band number than unrelated healthy control subjects in all three extracts (Fig. 1 A,C,E). In order to consider the quantitative intensity of reactivities, we further calculated principal components from the measured density quantitations of all bands detected in the three extracts, respectively. The resulting first principal components fitted 38% (HEp2-cytoplasm), 22% (HEp2-nucleus) and 17% (brain) of the respective total variance, and their factor loads were generally positive, so that factor-1 (F1) scores could be interpreted as representations of fitted overall reactivity. F1 distributions showed principally the same properties as band numbers (Fig. 1 B,D,F), with F1 scores significantly discriminating unaffected relatives from unrelated control subjects in terms of reactivity to both HEp2 extracts, while not to in respect to brain proteins. We finally performed quantitative ELISAs for plasma IgG binding to dsDNA, Ro60/SS-A, Sm and nRNP autoantigens, where samples were assayed with identical total protein concentration as in the immunoblots. Also in these antigen-specific assays, the unaffected relatives gave results intermediate between SLE patients and unrelated healthy subjects. Their difference to the control group was significant for IgG anti-dsDNA and anti-Sm (Fig. 1 G,H,I,J). Anti-dsDNA and anti-Ro60/SS-A assays were already reported for a subset of the present samples in our previous heritability study [10].

**Table 1:** Sample characterization.

	SLE Patients	Unaff. Relatives	Controls
Total	128	215	140
Gender	116 F, 12 M	129 F, 86 M	74 F, 66 M
Age (Median)	14–78 (37)	6–84 (38)	19–65 (43)

### 2. Positive correlations between autoantibody reactivity and frequencies of circulating active regulatory T-cells in unaffected relatives

**Figure 2:**
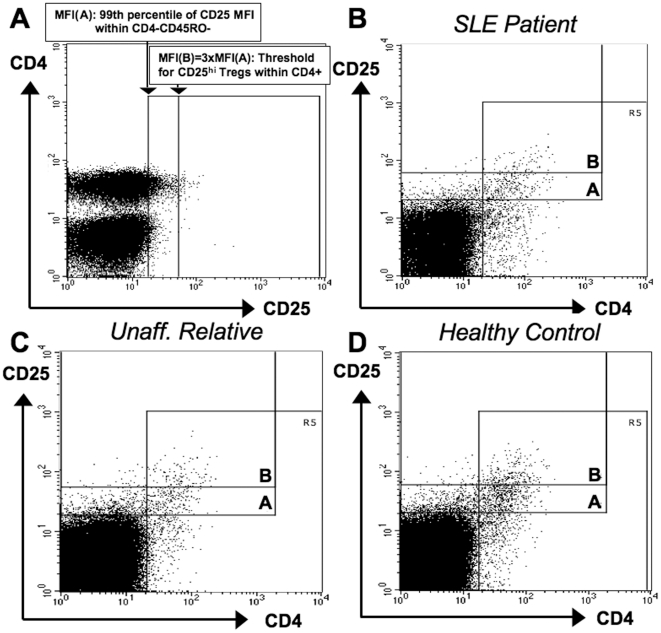
Systematic gating for CD25^bright^ aTregs. A. First, in order to specify the upper limit of CD25 staining in conventional T-cells for each sample, the ninety-ninth percentile of PE/Cy5 fluorescence intensity within CD4^−^CD45RO^−^ cells (a population containing no Tregs) was determined as gate A. CD4^+^CD25^bright^ aTregs in the same sample were then quantified as those CD4^+^ cells that exceeded this value at least three-fold (gate B). B–D. The CD25^bright^ gate (B) contains high proportions of Foxp3+ cells in samples from SLE patients, unaffected relatives and healthy control subjects. As exemplified by an active SLE patient (panel B), an unaffected relative (panel C) and a healthy control subject (panel D), the CD25^bright^ gate B defined as 3xMFI(gate A) regularly contained 70–90% Foxp3+ cells within CD4+ lymphocytes when additional samples were stained for the same markers as previously but now in combination with intracellular Foxp3 staining.

**Figure 3:**
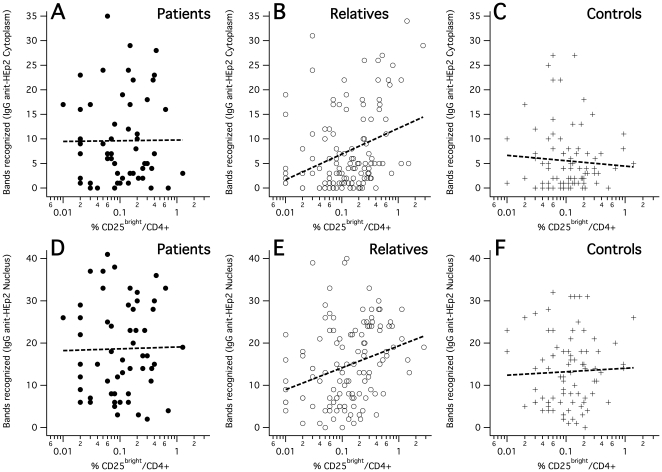
Correlations between CD25^bright^/CD4^+^ aTreg frequencies and immunoblot band numbers. A–C: Bands recognized in immunoblots of HEp2-cytoplasmic proteins by SLE patients, unaffected relatives and unrelated control subjects. D–F: Bands recognized in immunoblots of HEp2-nuclear proteins. Regression lines represent linear regression.

**Figure 4:**
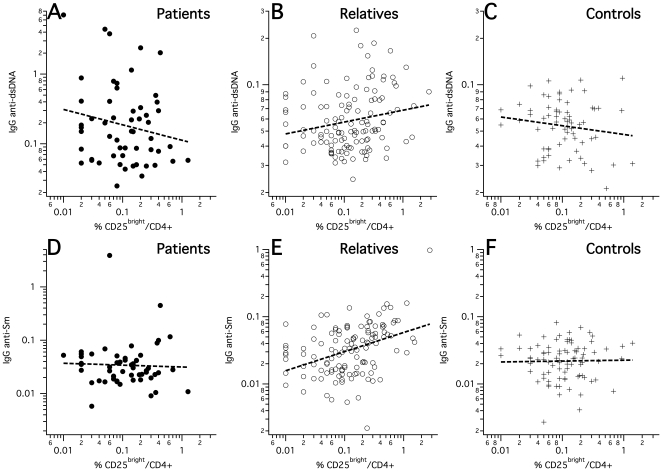
Correlations between aTreg frequencies (CD25^bright^/CD4^+^) and quantified SLE-associated specific autoreactive IgG. A–C: IgG anti-dsDNA. D–F: IgG anti-Sm. Regression lines represent linear regression.

**Figure 5:**
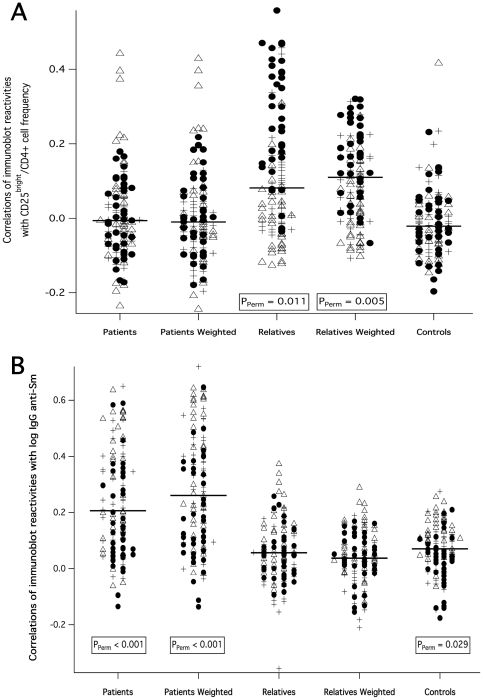
Unweighted and family-weighted correlation coefficients. Distributions of unweighted and family-wighted correlation coefficients of CD25^bright^/CD4^+^ aTreg frequencies (A) and log-transformed IgG anti-Sm (B) with all 130 single immunoblot reactivities are shown within SLE patients, unaffected relatives and unrelated controls, respectively. Reactivities to cytoplasmic bands are indicated by filled circles, anti-nuclear reactivities by triangles and anti-brain reactivities by crosses. Annoteted P-values were calculated by the described permutation test.

In a major subset of the subjects studied for autoantibodies (56 SLE patients, 123 unaffected relatives and 87 healthy controls), we parallelly stained peripheral blood mononuclear cells (PBMC), freshly isolated together with the plasma, for flow-cytometric analysis in order to quantify active regulatory T-cells (aTregs). Results from these experiments have been published earlier in the context of a genetic study [29]. Following the way how human Treg subsets were recently functionally tested and their activity validated on the basis of differential surface CD25 density [11], we quantified aTregs as CD25^bright^ within CD4^+^ T-cells. In order to consider the frequency of circulating aTregs in an unbiased way, we used an objectively defined threshold criterion (see methods). We particularly made sure by confirmatory stainings of 15 SLE patients, 45 unaffected relatives and 54 controls with combined CD25 and intracellular Foxp3 that the used CD25^bright^ gate regularly represented highly enriched Foxp3+ cells in all three subject groups (81±8% in SLE patients, 79±11% in relatives and 74±13% in controls, also see methods and Fig. 2 for representative cytometric profiles). Differences in the resulting aTreg frequencies between SLE patients, unaffected relatives and unrelated controls were not significant. However, aTreg cell frequencies had highly significant positive correlations with the band numbers of IgG autoantibodies reactive toward HEp2 nuclear and cytoplasmic proteins when calculated within the SLE-unaffected relatives group (Fig. 3, Table 2), in contrast to SLE patients or unrelated control subjects. Similarly significant positive correlations exclusively within the relatives group were also found between aTreg frequencies and all measured SLE-associated plasma IgG autoantibodies: anti-dsDNA (within unaffected relatives: *R_Spearman_* = +0.23 [*P* = 0.01]), anti-SSA (*R_Spearman_* = +0.24 [*P* = 0.007]), anti-Sm (*R_Spearman_* = +0.40 [*P* = 8E-6]), and anti-nRNP (*R_Spearman_* = +0.31 [*P* = 0.0006]), but not IgM anti-dsDNA (Fig. 4, Table 2). To confirm this result, we further calculated within-group correlations with each of the quantitative densities of the 130 detectable immunoblot bands separately, and tested the group-wise medians of the resulting 130 correlation coefficients against respective permutation-based null distributions (Fig. 5A). A difference from zero was only detected for the unaffected relatives group. These correlations became even stronger when using weighted correlations, considering each family with equal weight in order to avoid bias due to overrepresentation of families with many relatives. The highest correlation coefficients clearly came from IgG reactivities to HEp2-cytoplasmic antigens.

**Table 2:** Group-wise Spearman rank correlations with CD25^bright^/CD4^+^ frequencies.

Within Group	SLE Patients		Unaff. Relatives		Controls	
	*R_Spearman_*	*P_Spearman_*	*R_Spearman_*	*P_Spearman_*	*R_Spearman_*	*P_Spearman_*
Recognized IgG bands to HEp2-cytoplasmic proteins	−0.03	0.826	**+0.34**	**0.0002**	+0.03	0.768
Recognized IgG bands to HEp2-nuclear proteins	+0.07	0.622	**+0.34**	**0.0002**	+0.06	0.597
Recognized IgG bands to human brain proteins	+0.05	0.732	+0.17	0.061	−0.11	0.318
IgG anti-dsDNA	−0.17	0.217	**+0.23**	**0.011**	−0.10	0.399
IgG anti-Ro60/SSA	−0.17	0.220	**+0.24**	**0.007**	−0.05	0.654
IgG anti-Sm	−0.11	0.400	**+0.40**	**0.000008**	+0.02	0.827
IgG anti-nRNP	−0.06	0.648	**+0.31**	**0.0006**	+0.04	0.709
IgM anti-dsDNA	+0.11	0.407	+0.08	0.364	−0.07	0.543

For comparison, we analyzed analogous group-wise correlations of SLE-associated specific IgG with the same panel of immunoblot reactivities. In contrast to those of the aTreg frequencies, correlations calculated for IgG anti-Sm were very strong in the patients group (*R_median,weighted_* = +0.26; *P_Perm_*<0.001) but insignificant for unaffected relatives (Fig. 5B), despite the fact that the latter had an almost equal reactivity to Sm. Similar median correlations as for anti-Sm were found for IgG anti-nRNP (significant only for patients: *R_median,weighted_* = +0.17; *P_Perm_*<0.001), while IgG anti-Ro60/SSA rather behaved like aTreg frequencies and showed significant positive median correlations only in the relatives (*R_median,weighted_* = +0.11; *P_Perm_* = 0.002) and also in the control group (*R_median_* = +0.10; *P_Perm_* = 0.001).

In summary, we interpreted these findings so that unaffected relatives were likely to bear a particular regulatory capacity that involved compensatory expansion of aTregs in parallel to their individual extent of multispecific IgG autoantibody production, including SLE-related and other specificities. These aTregs could be triggered either by autoantibodies or by signals from B-cells and/or T-helper cells involved in their production. The SLE patients' multispecific autoreactive IgG repertoire, in contrast, was not obviously related to aTregs but instead strongly related to SLE-specific IgG such as anti-Sm and anti-nRNP, in consistence with the epitope spreading concept [5], [6].

### 3. Coreferentiality: a new approach to functional relatedness

Correlation does not necessarily reflect functional relatedness, neither is it always observed when two variables are functionally related, i.e., when they have impacts on the same effect. In order to address the functional relations between IgG autoantibodies and aTregs in our unaffected relatives, we developed a new approach to detect functional relatedness irrespectively of direct correlations. Self-reactive immune repertoires capable of triggering aTregs in unaffected relatives could be either natural or originating from the recognition of SLE-related autoantigens, by crossreactivity or epitope spreading. In both cases they likely included a broad and diverse spectrum of specificities, i.e., a substructure inside self-reactive repertoires. We could, therefore, expect that the more a specific measurable parameter was functionally related to the mechanisms that shaped the described relation between aTregs and self-reactive IgG, the more it would relate to the same substructure inside the entire self-reactive repertoire as aTreg frequency did. This could be tested by comparing how two parameters (here: aTreg frequency and a second parameter to be tested) respectively related to a standardized multivariate dataset representative for self-reactive immune repertoires, e.g., our 130 quantitatively standardized immunoblot IgG reactivities. A test criterion for specific relatedness between the two parameters could then be formulated as follows: were they correlated with the same elements inside the reference system in the same way, i.e., did they behave *coreferentially* toward it ? Mathematically, coreferentiality between two test parameters can be formulated as a second-order correlation between two vectors containing correlation coefficients of each respective test parameter with the vector of the multiple reference variables [40], and its statistical significance tested by comparison to a null distribution generated by data permutations. Using a test that is principally robust against direct correlations through parallel permutation of the two test parameters, we have found a remarkable power of this method in simulated data [40]. This allows us here to interpret coreferentiality as an independent indicator of relatedness.

**Figure 6:**
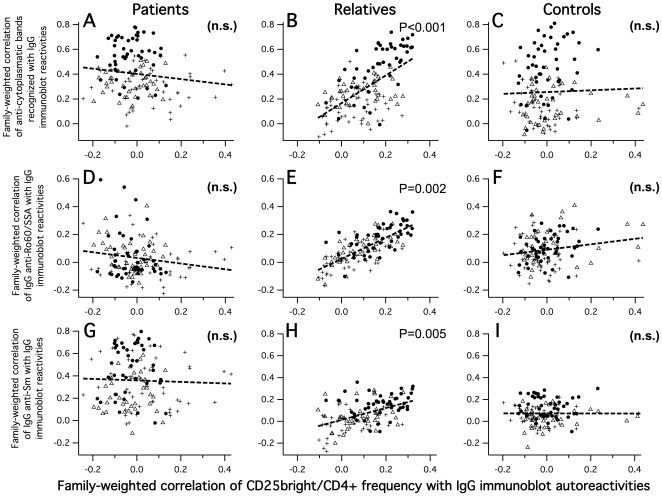
Group-wise coreferentialities with aTreg frequencies, in respect to 130 IgG immunoblot reactivities as reference data. A–C: Coreferentiality between CD25^bright^/CD4^+^ aTreg frequencies and IgG anti-HEp2-cytoplasmic immunoblot band numbers. D–F: Coreferentiality between aTreg frequencies and log-transformed IgG anti-Ro60/SSA. G–I: Coreferentiality between aTreg frequencies and log-transformed IgG anti-Sm. Reactivities to cytoplasmic bands are indicated by filled circles, anti-nuclear reactivities by triangles and anti-brain reactivities by crosses.

We first wanted to know if particular repertoire substructures were functionally related to aTregs. Were proteins originating from the three different antigen sources (nuclear, cytoplasmic, brain) equally involved in our hypothesized feedback regulation or not ? The coreferentiality criterion was appropriate to answer this, formulated as follows: would the same among the 130 immunoblot reactivities that correlated most positively with aTreg frequencies also correlate most positively with the diversity, i.e., the recognized band number, of IgG reactive toward each given antigen source, and vice versa ? Coreferentiality with aTreg frequencies was tested accordingly, separately within the groups of SLE patients, unaffected relatives and unrelated healthy controls. Weighted first-order correlations were used in order to correct for unequal representations of families. While no significant coreferentiality was found for any of our three band number counts within patients or unrelated control subjects, it was highly significant within the unaffected relatives group, but exclusively for the diversity of IgG to HEp2-cytoplasmic proteins. Analogously, we asked whether aTreg frequencies were coreferential with SLE-associated specific IgG autoantibodies. Once again, we found no significance within SLE patients or the unrelated control group, while log-transformed IgG anti-dsDNA, anti-SSA, and anti-Sm measures showed significant coreferentiality within the unaffected relatives (Table 3, Fig. 6). We did not detect ‘bystander coreferentiality’ [40] (also see methods) in any significant test. This demonstrated that the coreferentiality approach was capable of revealing immune repertoire substructures. In fact, a particular self-reactive substructure appeared functionally related to T-cell regulation in unaffected relatives, involving the IgG recognition of diverse cytoplasmic proteins along with SSA/Ro60, dsDNA and also Sm - highly recognized by the relatives, but directly correlated with multispecific IgGs rather in the patients than the relatives group.

**Table 3:** Coreferentialities with CD25^bright^/CD4^+^ frequencies.

Within Group	SLE Patients		Unaff. Relatives		Controls	
	*R_C_*	*P_Perm_* *	*R_C_*	*P_Perm_* *	*R_C_*	*P_Perm_* *
Recognized IgG bands to HEp2-cytoplasmic proteins	−0.13	0.491	**+0.61**	**<0.001**	+0.04	0.792
Recognized IgG bands to HEp2-nuclear proteins	−0.14	0.498	+0.36	0.257	+0.23	0.232
Recognized IgG bands to human brain proteins	+0.20	0.375	+0.24	0.694	−0.04	0.794
log IgG anti-dsDNA	−0.12	0.589	**+0.59**	**0.019**	−0.03	0.904
log IgG anti-Ro60/SSA	−0.16	0.314	**+0.74**	**0.002**	+0.18	0.185
log IgG anti-Sm	−0.03	0.848	**+0.48**	**0.005**	+0.00	0.987
log IgG anti-nRNP	−0.09	0.635	+0.30	0.212	+0.13	0.401
log IgM anti-dsDNA	−0.17	0.557	+0.34	0.055	+0.30	0.066

* Significance according to permutation test (see methods). Effects of bystander coreferentiality were checked for all significant tests (see methods), but rates of simulated bystander data reaching the respective test significance never exceeded 5%.

### 4. Genotype coreferentiality: an approach to the role of IL-2-CD25 interaction

Next we wanted to apply the coreferentiality approach to interpret the role of a molecular mechanism with evident relevance for T-cell regulation and likely for SLE pathology: the interaction of IL-2 with CD25. If our above interpretation was correct and CD25^bright^ aTregs were compensatorily expanded in unaffected relatives and exerted a regulatory feedback function, we could readily expect that this effect depended on IL-2-CD25 interaction. To test if a functional context of IL-2, CD25 and aTreg effects in our unaffected relatives was related to a particular immune repertoire structure, we chose an approach based on the obvious fact that IL-2 and CD25 are closely functionally related. Therefore, if the coreferentiality approach was valid and sufficiently powerful, we could expect to detect coreferentiality. Thus, would the respective functionalities of IL-2 and CD25 be coreferential in respect to our multivariate reference system of autoreactive IgGs, indicating that IL-2-CD25 interaction influenced the self-reactive IgG repertoire in a defined manner ? And if so, was this effect related to T-cell regulation ? Unfortunately, IL-2 itself largely escapes meaningful measurement due to its local effect and short life span. Also the physiologic functionality of CD25 in lymphatic organs is not clearly reflected by measures in conditions where they are feasible, namely the use of peripheral blood cells. In contrast, genetic variation can easily and accurately be assessed, and does not vary with measurement conditions. Polymorphisms with likely functional effects have indeed been described for both the loci *IL2* and *IL2RA* encoding CD25 (see discussion). Since genotypes are furthermore individually invariant and independent of phenotypic effects, coreferentiality between genetic effects could be expected to be particularly robust. Hence, we first aimed to find out whether *IL2* genetic variation behaved coreferentially in respect to IgG repertoires with genetic variation in the *IL2RA* locus, independently of aTregs.

In the relatively small *IL2* locus, two SNPs have been most studied: *rs2069762*, located within the promoter region and associated to differences in IL-2 production by cultured cells [41], and *rs2069763*, synonymous-coding in the first exon, the minor allele of which was recently found protective against SLE in a Chinese population [42]. We typed these two and six other SNPs covering the *IL2* locus and the 5′ and 3′ flanking regions (see LD map, Fig. S2). Among the additional six SNPs, two had low minor allele frequencies below 5%, and two others were in strong LD with *rs2069763*, while the remaining two, *rs1479924* and *rs11575812*, formed a new linkage group in almost complete LD (*R^2^* = 0.94) although located 5′ and 3′ of the coding region, respectively. We further found that for *rs11575812*, *rs2069762* and *rs2069763*, each respective minor allele very likely represented an independent locus-spanning haplotype (see discussion), which is compatible with the fact that *rs11575812* and the strongly linked *rs1479924* are located at opposite sides of the locus. To cover the much larger *IL2RA* locus, we typed 25 SNPs (Table S1), including some for which significant association with type-1 diabetes has been reported [43]. We first tested coreferentiality in unaffected relatives, for the scores of *rs11575812*, *rs2069762* and *rs2069763*, respectively, with all 25 *IL2RA* SNPs simultaneously by data permutations. Surprisingly, we found a particularly strong coreferentiality for *rs11575812* (|*R_C_*|*_max_* = 0.6; *P_Perm_* = 0.044).

### 5. A CD25 genetic-effects model achieved by optimized *IL2*-*IL2RA* coreferentiality in the relatives group is plausible in several respects and group-independent

The finding that functional effects of genetic variation in the *IL2* and *IL2RA* loci indeed parallelled each other in the unaffected relatives in their relations to self-reactive repertoires, together with the evidence for strong immune regulation in this group, prompted us to use coreferentiality as a modeling tool. Since transcriptional regulation of IL-2 is known to be complex and specifically affected by SLE [17], we particularly sought to derive a model for functional genetic effects in the *IL2RA* locus. We did this by maximizing the coreferentiality between two functions representing linear combinations of (a) the three principal *IL2* SNPs (*rs11575812*, *rs2069762*, *rs2069763*) and (b) five representative *IL2RA* SNPs (see methods). The optimized model reached a coreferentiality of *R_C max_* = 0.77, maximized by functions with the following normalized coefficients for (a) *IL2* and (b) *IL2RA* SNP scores:

−0.88[*rs11575812*]+0.03[*rs2069762*]−0.47[*rs2069763*];
−0.33[*rs791589*]+0.15[*rs706778*]+0.51[*rs7073236*]−0.77[*rs10795791*]+0.14[*rs11594656*].

In order to test whether the observed degree of optimized coreferentiality was meaningful or could also be achieved accidentally, we optimized 50 further models with the same method but using random data permutations instead of our real data (the usual 1,000 permutations being impossible to perform here due to computational time). In this null distribution of models, only two out of 50 gave maximized coreferentialities reaching the value of 0.77 found for the true data, corresponding to an empirical significance of *P* = 0.04. Thus, the derived model was likely non-random and meaningful, and we proceeded to analyze its properties.

**Figure 7:**
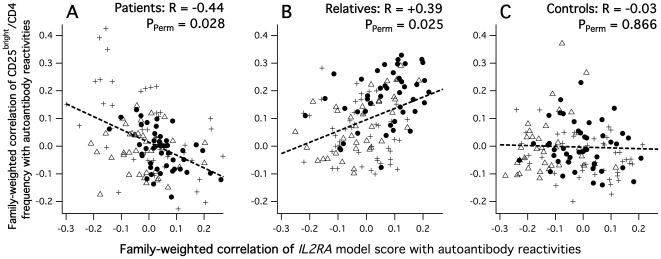
Group-wise coreferentialities between CD25^bright^/CD4+ aTreg frequencies and *IL2RA* genetic-effects model scores. A: SLE patients, B: unaffected relatives, C: unrelated controls. Reactivities to cytoplasmic bands are indicated by filled circles, anti-nuclear reactivities by triangles and anti-brain reactivities by crosses.

In order to score the respective functional *IL2* and *IL2RA* genetic effects according to this optimization, we calculated both model scores for all typed unaffected relatives from functions (a) and (b), respectively. *IL2* and *IL2RA* model scores were not significantly correlated with each other (*R_Spearman_* = 0.08; *P* = 0.26), excluding a trivial interpretation of the achieved coreferentiality by an artificial score correlation. Next, we wanted to know whether the resulting *IL2RA* model was compatible with an effect related to T-cell regulation as it was suggested above. Cytometric aTreg frequencies, which had not been used to derive the *IL2-IL2RA* effects model, could in fact now be used to test this: correlated with self-reactive IgG and coreferential with repertoire substructures as shown above, were they also coreferential with the independently modeled *IL2RA* genetic effect in the unaffected relatives group ? Indeed, we found a significant coreferentiality of *R_C_* = +0.39 (*P_Perm_* = 0.025, see also Fig. 7) with no bystander effect. This supported the meaningfulness of the model and also allowed to interpret its score resulting from function (b): if CD25 had a parallel functional effect with aTregs on autoantibody repertoires, then given a positively signed *R_C_*, the model score indicated a positive effect on CD25 expression and/or functionality.

If our model was valid, the modeled genetic CD25 effects on autoantibodies with a role in immune regulation had to depend on specific antigen recognition by CD4^+^ T-cells. A straightforward way to test this was whether our model was also related to genetic variation in MHC class II molecules that shape the CD4^+^ T-cell repertoire. Therefore, we determined and scored seven major HLA-DR types. Tested cumulatively, there was significant coreferentiality with our *IL2RA* model score within unaffected relatives (Table 4), but not with aTreg frequency or any of our autoantibody measures (not shown). Univariately, HLA-DRB1*04 and DRB1*13 were significant for positive, and DRB1*01 for negative coreferentiality with the *IL2RA* model score, suggesting that these HLA types had effects on autoreactive IgG in the unaffected relatives resembling that of stronger or weaker CD25 functionality. We also analyzed analogously whether the modeled genetic CD25 effect was coreferential with specific SLE-associated autoantibodies that had shown significant correlations and coreferentiality with aTreg frequencies. This, however, was not the case: coreferentiality (as well as direct correlations, not shown) of the model score with specific IgG measures or the IgG band number detected to HEp2-cytoplasmic proteins remained insignificant. In contrast, it was significant with the IgG band number toward HEp2-nuclear proteins (Table 4).

**Table 4:** Coreferentialities of the *IL2RA* model score with other parameters.

Within Group	Unaff. Relatives		SLE Patients		Controls	
	(optimized)					
	*R_C_*	*P_Perm_*	*R_C_*	*P_Perm_*	*R_C_*	*P_Perm_*
Frequency CD25^bright^/CD4^+^	**+0.39**	**0.025**	**−0.44**	**0.028**	−0.03	0.866
*CD247* (CD3Z) All typed SNPs*	0.57	0.118	**0.51**	**0.041**	**0.57**	**0.023**
log IgG anti-dsDNA	−0.03	0.894	+0.36	0.112	+0.09	0.548
log IgG anti-Ro60/SSA	+0.03	0.851	+0.21	0.247	+0.11	0.535
log IgG anti-Sm	+0.40	0.156	+0.15	0.379	**+0.37**	**0.014**
log IgG anti-nRNP	+0.34	0.099	+0.22	0.231	**+0.37**	**0.003**
log IgM anti-dsDNA	+0.05	0.757	**+0.49**	**0.010**	−0.17	0.237
Recognized IgG bands to HEp2-cytoplasmic proteins	+0.14	0.574	+0.06	0.749	+0.28	0.067
Recognized IgG bands to HEp2-nuclear proteins	**+0.36**	**0.030**	+0.32	0.084	−0.28	0.066
Recognized IgG bands to brain proteins	−0.04	0.947	−0.06	0.696	+0.29	0.057
HLA-DRB1*01 Alleles	**−0.56**	**0.007**	+0.18	0.416	*(not typed)*	
HLA-DRB1*03 Alleles	+0.25	0.282	+0.04	0.817	*(not typed)*	
HLA-DRB1*04 Alleles	**+0.62**	**0.006**	+0.00	0.978	*(not typed)*	
HLA-DRB1*07 Alleles	−0.37	0.097	−0.18	0.372	*(not typed)*	
HLA-DRB1*11 Alleles	+0.33	0.129	+0.37	0.128	*(not typed)*	
HLA-DRB1*13 Alleles	**+0.56**	**0.035**	+0.09	0.593	*(not typed)*	
HLA-DRB1*15 Alleles	−0.09	0.838	−0.01	0.971	*(not typed)*	
All HLA-DRB1 types*	**0.62**	**0.027**	0.37	0.338		

* Test for multiple parameters, on maximal absolute coreferentiality |*R_C_*|_max_ (see methods). Significant tests, except those for multiple SNPs, were also checked for the effect of bystander coreferentiality (see methods), but no such effect was detected.

*IL2-IL2RA* coreferentiality maximization in the patients group, in contrast to the relatives, yielded a low value (0.57) and was therefore not followed. However, while the transcriptional control of IL-2 is known to be altered in SLE pathology [17], there is no such indication for CD25, so that a valid model for functional genetic effects likely represented them in all individuals. If this was the case for our *IL2RA* model optimized for the unaffected relatives, it should be applicable also beyond that group. Indeed, model scores calculated with function (b) from genotypes of patients or unrelated controls showed the same principal relatedness with the dominant *IL2* SNP *rs11575812*: coreferentiality (*R_C_* = −0.66 for the unaffected relatives) was equally signed and reached statistical significance when calculated within patients (*R_C_* = −0.37; *P_Perm_* = 0.033). It was also equally signed in the control group (*R_C_* = −0.11) although not significant. Next, addressing the relation of our *IL2RA* model with TCR-dependent cell activation, it was not coreferential with HLA-DR types in SLE patients as it had been in the unaffected relatives (Table 4). For other reasons, we had typed our subjects for 24 SNPs covering the *CD247* locus (Table S1) that encodes the CD3ζ chain, the major TCR signal transducer that is characteristically downregulated and replaced in SLE T-cells [44], which is a causal factor for transcriptional IL-2 repression [45]. Testing our *IL2RA* model for coreferentiality with the cumulated *CD247* SNPs, we found similarly high locus-wise maximal coreferentialities for all three groups (Table 4), significant for patients and for the control group as well as when calculated in all three groups merged (|*R_C_*|*_max_* = 0.49; *P_Perm_* = 0.040). We also found significant positive coreferentialities with IgG anti-Sm and anti-nRNP in the control group. If our *IL2RA* model was generally representative for *IL2RA* functional genetic effects and functionally related to CD3ζ effects, we further expected that an *IL2RA* genetic effects model independently optimized for *CD247-IL2RA* coreferentiality would be close to that derived by *IL2-IL2RA* coreferentiality optimization. Indeed, coreferentiality optimization within the unrelated controls, using the same five *IL2RA* SNPs as above and three *CD247* SNPs, led to an *IL2RA* model with almost identical coefficients, and scores that were more than 90% correlated with those of the initial *IL2-IL2RA* model in all three subject groups (Table 5).

**Table 5:** *IL2RA* model properties.

	Normalized coefficients for IL2RA SNP scores					Correlation coefficients of model scores with IL2RA-IL2 model			Optimized model *R_C_*
	*rs791589*	*rs706778*	*rs7073236*	*rs10795791*	*rs11594656*	Pat.	Rel.	Ctl.	
Model *IL2RA-IL2* in unaffected relatives	−0,328	+0,148	+0,514	−0,767	+0,135	-	-	-	0.77
Model *IL2RA-CD247* in unrelated controls	−0,311	+0,208	+0,545	−0,743	+0,103	0.96	0.95	0.96	0.67
Model *IL2RA-*Treg frequency in patients	−0,077	+0,224	+0,472	−0,484	+0,698	0.93	0.84	0.93	0.59

### 6. Modeling suggests inverse IL-2 effects in patients and unaffected relatives

Given the plausibility that our initial unaffected-relatives-optimized *IL2RA* model represented the same *IL2RA* functional genetic effects in all subjects, we finally tested whether it was coreferential with cytometric aTreg frequencies in the SLE patients group. Surprisingly, we found a significant negative coreferentiality of *R_C_* = −0.44 (*P_Perm_* = 0.028), inversely signed in respect to that found for the unaffected relatives (Fig. 7, Table 4), and with no bystander effect. To confirm whether inverse *IL2RA*-aTreg coreferentialities in patients and unaffected relatives were a valid result, we optimized a second independent *IL2RA* model for coreferentiality between the five initially used *IL2RA* SNP scores and aTreg frequency in the SLE patients group, and found that this *IL2RA* model was also similar to the initial one as was that optimized with *CD247* SNPs in controls (Table 5).

## Discussion

We describe here that unaffected relatives of SLE patients, who frequently presented SLE-related IgG autoantibodies, showed remarkable and consistently significant positive correlations of their circulating active Treg frequencies with SLE-associated IgG specificities as well as with reactivity band numbers directed toward immunoblot-separated autoantigens. These positive correlations were unique for the group of unaffected relatives, and neither found in SLE patients nor in unrelated control subjects, suggesting that they were a reflection of a compensatory feedback mechanism that contributed to the prevention of overt disease in unaffected relatives who shared genetic susceptibility factors for SLE-associated autoantibody diversification. Such a mechanism could be triggered by either certain autoantibodies themselves, or by T-helper cells involved in their generation, and lead to an expansion of aTregs with the capacity of controlling potentially pathogenic effects. This is consistent with mouse lupus models, where disease manifestation was found preceded by reduced Treg numbers [46], accelerated by Treg depletion [47], and delayed or prevented by complementation with *in vitro* expanded Tregs extracted from young mice [48], [49]. In human families affected with SLE, healthy family members with elevated SLE-type autoantibody reactivity were repeatedly reported [8]–[10], [50], without indication of being at high risk to develop SLE. These autoantibody-positive unaffected relatives, however, were so far systematically studied only for heritability, which was principally confirmed for the autoantibody trait but remains unclear in respect to specificities [8]. Healthy individuals with high levels of virtually all SLE-associated autoantibody specificities were also found in the general population, although at low frequencies [51], [52]. A mechanism of active Treg-mediated SLE compensation in these individuals, as we suggest here, favors therapeutic perspectives to either restore this compensation or prevent SLE at an early stage by Tregs or Treg-supporting therapies.

We did not stain for Foxp3 in this study since intracellular anti-Foxp3 staining, now regularly used to characterize Tregs, was not available when the experiments were performed. Neither were auxiliary Treg markers such as CD127 known. However, the way we have studied aTregs appears accurate also in respect to current knowledge, particularly since recent literature makes it more and more likely that CD25 (gated with a high threshold) is a more important marker for active Tregs than Foxp3: it has been well demonstrated that a relevant subset of peripheral human CD4^+^Foxp3^+^ cells that is increased in SLE and most characteristically shows low or absent (but not bright) surface CD25, does not consist of functionally active Tregs [11], [25]–[28], [53], [54]. Our objectively defined gate for CD25^bright^ aTregs efficiently excludes this population, thus can be said to use CD25 as the Treg activity marker as which the most recent literature characterizes it. At the same time our aTreg gate enriched Foxp3^+^ cells sufficiently and to similar extents in the three groups studied, as confirmatory Foxp3/CD25 stainings show (see methods, Fig. 2 and Fig. S3). Particularly, there was no indication of increased contamination of the gated CD25^bright^ cells by Foxp3^−^ T-cells in SLE patients that could theoretically occur in presence of disease-related Foxp3^−^ T-cell activation, which was once reported especially for patients with highly active disease [55]. Also other studies that we have meanwhile performed indicate no significantly increased surface CD25 in CD4^+^Foxp3^−^ cells of our SLE patients when compared to controls (unpublished data).

IL-2 is commonly regarded as the most important maintenance factor for Tregs [12]. Deficiency for either IL-2 or CD25, which is the specific element of the high affinity IL-2 receptor that also characterizes human aTregs, results in impaired Treg functionality and autoimmunity in mice [13]–[15]. Dysregulation and relative transcriptional repression of IL-2, in turn, have been well documented in SLE [17]. Therefore it was straightforward for us to address the question whether the hypothesized compensatory mechanism in unaffected relatives was IL-2-related. To study this, we used a novel approach, coreferentiality, to derive a model of IL-2-mediated functional effects depending on genetic variation in the *IL2RA* locus. The properties of the derived and empirically validated model suggested that (i) the IgG autoreactive repertoire was indeed significantly influenced by IL-2-CD25 interaction in the unaffected relatives, and (ii) aTreg frequency in this group was related to a similar substructure in the autoantibody repertoire as was the modeled IL-2-CD25-mediated genetic effect: both were coreferential, thus likely functionally related. Applied to SLE patients, the same *IL2RA* genetic-effects model surprisingly showed an inverse coreferentiality with aTreg frequency, indicating that the modeled IL-2-mediated effects were functionally *opposed* to aTreg effects. This is most easily explainable by the notion that IL-2, in the condition of manifest SLE, favored T-effectors rather than aTregs.

This explanation is consistent with the known transcriptional IL-2 repression in SLE, which, however, was so far rather seen associated with altered effector cell activation. In any case it appears to be part of a systemic T-cell pathology that includes aspects of both hypo- and hyperactivity, i.e., more rapid but dysregulated and less sustainable responses [44]. Altered or deficient Treg functionality is in fact likely to be an important and early aspect of this dysregulation. This is supported by the now well-documented characteristic reduction or even absence of surface CD25 on likely dysfunctional Tregs in SLE [26], [27], which was indicative for their known reduced suppressive function [24] and ‘dissociated’ from the often proportionally increased Foxp3 expression in SLE [28], [53]. Furthermore, in line with similar data from diabetic NOD mice [56], a recent report demonstrates that lupus pathogenesis in NZB/W F1 mice could be either accelerated by IL-2 neutralization or impeded by IL-2 complementation [18], and proposes a pathogenesis model driven by interdependent Treg and IL-2 deficiencies. However, relative Treg deficiency rather preceded IL-2-mediated effects in that study. The opposed effects of IL-2 and aTregs in SLE patients suggested by our model support the same notion that reduced IL-2 may not be directly coincident with deficient regulation. Our present results also point to separate factors contributing to Treg functionality in unaffected relatives. Effects of *IL2RA* genetic variation in this group, although related to the aTreg effects on autoantibodies, primarily concerned an autoantibody repertoire that was clearly dependent on HLA-DR types, focused on nuclear rather than cytoplasmic targets, and showed no evident relation to SLE-associated specific IgG. Unaffected relatives' aTreg frequencies, however, were related to an autoantibody profile with no evident HLA-DR dependency, focused on cytoplasmic targets and also strongly coreferential with the presence of SLE-associated specific IgG. This suggests that the hypothesized compensatory mechanism that lead to the initially observed positive correlations between autoantibodies and aTreg frequency was not only driven by the IL-2-mediated repertoire effects, but by an additional factor that was related to the recognition of SLE-specific antigens and less HLA-dependent. In summary, it appears plausible that strong IL-2-mediated effects principally favor aTregs in immune reactions including natural self antigens but without an apriori relation to SLE autoantigens, and that the efficiency of this mechanism is a condition for SLE compensation in genetically susceptible but healthy individuals. Nevertheless, specific aTregs recognizing particular epitopes and expanded or induced in SLE autoantigen-specific immune reactions that also lead to the presence of SLE-type autoantibodies, may well control the potentially pathogenic effects of these reactions. SLE could become clinically manifest when this specific compensatory regulation breaks down. In the compensated state, IL-2 reduction or dysregulation might favor the loss of the specific aTregs. In their absence, however, IL-2 could directly trigger pathogenic effector functions. This could make potential IL-2 therapies a double-edged sword. It would, however, generally encourage efforts to develop new therapies stabilizing or even restoring the compensated state by supporting or expanding Tregs with particularly relevant specificities.

In the context of the studied *IL2* polymorphisms, the strongest genetic effect in our model was attributed to the 3′ SNP *rs11575812*, which was in almost complete linkage with the far 5′ *rs1479924*. To our knowledge, this linkage group has not been described or considered in the context of association studies so far. However, it showed a remarkable property that extends earlier observations. Thus, an earlier study of the two traditionally typed SNPs *rs2069762* (5′ promoter region, also referred to as −384 or −330) and *rs2069763* (1st-exon synonymous-coding, +114) had already noted that the haplotype consisting of the combined respective minor alleles was inexistent [57]. Having typed *rs11575812* together with these two SNPs, we can confirm and extend this observation: none of our 448 completely typed individuals had more than two minor alleles of the three SNPs combined, while 79% had exactly two. This indicates that in fact no haplotype with more than one minor allele existed, and that therefore each respective minor allele in these three polymorphisms represented an independent locus-spanning haplotype, an interpretation that also explains the strong linkage between *rs11575812* and the distant *rs1479924*. In this context, the fact that the haplotype defined by the *rs11575812* variant, which according to our model had the strongest functional effect, was not considered in the earlier studies of genotype-function relationships [41], [57], [58], might explain inconsistencies between them. Nevertheless, all found significant genetic effects. The only study including IL-2 protein quantification [41] reported that the presence of the *rs2069762* minor G allele resulted in increased IL-2 secretion by anti-CD3/CD28-stimulated T-cells, thus could be ascribed a relatively positive effect on IL-2 functionality. Our unaffected-relatives *IL2* model ascribes negative functional effects to the respective minor alleles of both *rs11575812* and *rs2069763*, but no effect to that of *rs2069762*. In the context of the interpretation of all three minor alleles as independent haplotypes, this is consistent with the previously described relatively positive effect of *rs2069762* G. These haplotype structures may have particular functional relevance since IL-2 is monoallelically expressed in single human T-cells [59], although not stably in expanded clones [60]. *IL2* is also since long suspected for association with various disease phenotypes, but it has to be taken into account that linkage in the *IL2* locus (including the discussed haplotype structure) extends to the neighboring *IL21* gene and therefore association does not necessarily reflect IL-2-mediated effects. Association of the *IL2-IL21* linkage region is in fact established with autoimmune diseases such as type-1 diabetes [61], psoriasis [62] and celiac disease [63], while it remains unconfirmed for multiple sclerosis where *rs2069762* had shown the strongest trend [64], [65]. Isolated association of *rs2069763* with SLE was reported for a Chinese population [42]. Also genetic variation in the *IL2RA* locus has an evident functional relevance, clearly demonstrated by its association with rheumatoid arthritis [66], type-1 diabetes [43] and multiple sclerosis [65]. The two latter studies each detected two separate genetic effects located 5′ or in the 5′ portion of the coding region, which is the same region where four of our five SNPs selected for the *IL2RA* model function were located (selected by their coreferentiality with *rs11575812*). Two had in fact the highest impacts in the resulting model (*rs7073236*, *rs10795791*). One of the two *IL2RA*-region SNPs suspected to be causal [43], *rs11594656*, actually had a high coreferentiality with *rs11575812* and was included in our model function, but turned out to have little impact in the resulting model (see also Table S1).

Perhaps the most interesting aspect of the present study is the coreferentiality approach by itself, which we formally describe in an accompanying publication [40]. Applying it and following the principle whether principally known genetic effects were related with similar substructures in a multivariate set of phenotype variables, we were able to derive a model of combined genetic effects that showed all signs of plausibility. This approach is new, and we are not aware of publications describing anything similar. Modeling the functionality of combined genetic effects is usually beyond the scope and power even of large genetic studies, while we have only worked with a moderate sample number. This makes it interesting to discuss the perspectives of our approach in respect to genetics. Here it does in fact represent a strategy of investigation that principally differs from convention, reverting priorities: traditionally, genetic study first aims at the revelation of risk loci independently of any functional hypothesis, only to be followed by functionality-guided approaches toward detected risk factors, either directly by experiments or indirectly by interpreting genetic associations and/or expression patterns in the context of pathway analyses etc. Conversely, we used genotypes independently of being strong risk factors or showing phenotype association to study functional hypotheses such as a role of a particular cytokine-receptor system (IL-2) or cell type (aTregs) in a defined physiologic context. Our results suggest that genetic information in human populations can indeed be indirectly used in this way to interpret functionality, without necessarily following the traditional strategies of genetic study that are not based on functionally defined hypotheses. Systematically applied, this may open a totally new perspective of physiologic modeling, particularly in contexts that are not easily accessible to direct experimentation. In this way, our approach also revives the concept of subphenotype analysis in genetics. We used autoantibody repertoires as a reference system in our study, but it appears very exciting to apply the same analytic approach e. g. to microarray-based genome-wide RNA expression data. The dimensionality and depth of this and other types of data available with high-throughput technologies could exceed our reference system by orders of magnitude, and if coreferentiality-based models on the basis of such reference systems are as capable of delineating modules of functionally related effects as they appeared to be in our study, this is expected to have great analytic power.

## Materials and Methods

### Sample

128 SLE patients, 215 unaffected relatives and 20 unrelated spouses from 61 SLE-affected families, as well as 120 healthy blood donors were studied. Patients met the revised 1997 American College of Rheumatology criteria for SLE (Hochberg, 1997). The study was approved by the Ethics Committee of the Portuguese Lupus Patients Association, and written informed consent was obtained from all participants. Collections took place 2001–2004. For analysis, blood donors and unrelated spouses were cumulated in the group of unrelated healthy control subjects. Age and gender distributions are shown in Table 1. At collection, in order to address possibly undiagnosed subclinical SLE in unaffected relatives, they answered ten simple questions [67]. However, no autoantibody reactivity measure described was positively correlated with the number of positively answered questions.

### Plasma preparation, protein quantification and quantitative ELISA

Peripheral blood was collected in 7,5 mL *Vacutainer* CPT tubes containing citrate (Becton Dickinson) and centrifuged. Plasma supernatants were aliquoted and stored at −20°C. Total protein concentrations were determined by the Biuret method in an automated system (Hospital de Santa Cruz, Lisboa). Quantitative ELISAs were performed as described [10]. Briefly, plates (Pierce) were coated overnight with purified Ro60/SS-A (ImmunoVision) or dsDNA (Sigma-Aldrich), blocked with PBS-Gelatin 1%, and incubated overnight at 4°C with test plasma diluted in PBS containing 1% gelatin and 0.1% Tween-20 (for dsDNA without Tween) so that the final total protein concentration was always 0,5 mg/mL. After washing, plates were incubated overnight with alkaline phosphatase-conjugated anti-human IgG (Sigma-Aldrich) diluted 1∶1000, reactivity revealed with *p*-nitrophenyl phosphate (Sigma-Aldrich) and 405 nm absorbance measured by an ELISA plate reader (BioRad). Quantification was obtained by fitting ODs to a standard curve present in duplicates on each plate, consisting of 12 serial dilutions of positive control sera (ImmunoVision). One unit of reactivity was defined as the equivalent of the highest control serum concentration, previously chosen to be below saturation level.

### Quantitative immunoblot

Quantitative immunoblot was essentially carried out according to [30], [32], with recent adaptations by us [38], [39]. Briefly, nuclei and cytoplasm of trypsinized cultured HEp2 cells were separated [68], and nuclei further purified by Ficoll 12.5–25% gradient ultracentrifugation. Nuclear and cytoplasmic preparations, as well as human brain tissue obtained from a deceased person without preceding brain disease, were mechanically homogenized and solubilized, separated by 10% SDS-PAGE, electrotransferred onto nitrocellulose membranes and blocked as described [32]. Membranes were then incubated with plasma samples diluted in PBS/0.2% Tween-20 so that the final total protein concentration was 1 mg/mL, using a cassette system (Immunetics, Cambridge, MA) with separate incubation slots allowing to assay 24 samples per membrane. On every membrane, there were two replicates of a general reactivity standard (a mixture of plasma samples reactive to many bands, prepared in large quantity) and a quality control (Fig. S1). Samples were randomly distributed over the membranes. Incubation was for 4 h at room temperature, followed by detection by alkaline phosphatase-coupled chain-specific rabbit anti-human IgG (Sigma-Aldrich) for 90 min at room temperature. After revelation with NBT/BCIP, membranes were densitometrically scanned (600 dpi, 8-bit linear grayscale), stained with colloidal gold (Protogold, BritishBioCell, Cardiff, GB) for total protein, and scanned again. As described [32], protein staining in the spaces between incubation slots was used to adjust migration scales within and between membranes. The resulting standardized scale was divided into sections around immunoreactivity peaks. Peak areas under the respective densitometric profiles above baseline level were calculated for each section and divided by the section length. These standardized densities were further subtracted by a background density defined for each section and membrane as the 25^th^ percentile of all samples assayed on the same membrane (after verification that no visible reactivity band was recognized distinguishably from background by >75% of the samples), and normalized between membranes by division by the average standardized density of all sections of the two reactivity standard replicates present on each membrane. Given the random distribution of samples over the membranes, this standardization could be tested for possible artefacts by membrane-wise comparison, and no significant difference was detected between the respective 24 membranes blotted with the same extract (Kruskal-Wallis test).

Quantitative immunoblot assays in this study were adjusted for equal total plasma protein and not for total IgG as it was done in earlier studies with this method that had aimed to characterize naturally autoreactive repertoires and accordingly favored a proportional representation. Since, in contrast, this study aimed at representing and analyzing absolute levels of specific IgG as conventional serology does, total plasma protein levels were used for adjustment, which largely represent plasma albumin and not the individual variations in total circulating IgG.

### Flow cytometry

Peripheral blood mononuclear cells (PBMC) were isolated using Vacutainer CPT™ tubes (Becton Dickinson). 1×10^6^ cells were fixed in 2% paraformaldehyde, washed twice in PBS containing 2% fetal calf serum, and incubated for 20 min at 4°C with the optimal dilution of each conjugated anti-human mAb (CD4-FITC, CD31-PE, CD25-PE/Cy5 and CD45RO-APC, all from Becton Dickinson). Cells were washed again and fluorescence intensity measured using a FACScalibur flow cytometer and the CellQuest™ software. As a quantitative readout of aTregs, without Foxp3 staining that was not available when this study was done, but in consistency with their recent characterization [11], frequencies of CD25^bright^ within CD4^+^ cells were measured as follows. First, in order to specify the upper limit of CD25 staining in non-CD25-expressing cells for each sample, the ninety-ninth percentile of PE/Cy5 fluorescence intensity within CD4^−^CD45RO^−^ cells was determined. CD4^+^CD25^bright^ aTregs in the same sample were then quantified as those CD4^+^ cells that exceeded this value at least three-fold (Fig. 2).

Later, when intracellular Foxp3 staining was available, we combined it (according to the instructions of ebioscience, the manufacturer of the anti-Foxp3 staining kit) with CD4 and CD25 surface staining. The evaluation of this combination in 15 SLE patients, 45 unaffected relatives and 54 blood donor controls confirmed that the described aTreg gate (Fig. 2) regularly contained similarly high proportions of Foxp3^+^ cells in all three groups: 81±8% (SLE patients), 79±11% (relatives) and 74±13% (controls). Particularly, the SLE patients did not show any indication for increased contamination of gated CD25^bright^ by Foxp3^−^ cells, which can be seen in the individual cytograms of our 15 confirmatory stainings (Fig. S3).

### Genotyping

Genomic DNA was extracted by standard methods. SNP genotyping was performed on the Sequenom™ platform using multiplexed amplification followed by mass-spectrometric product separation. For the *IL2* SNPs *rs2069762* and *rs2069763*, some samples that did not call were also typed by RFLP as described [69], and the genotype information complemented accordingly. All SNPs used were in Hardy-Weinberg equilibrium (*P*>0.01), indicated in Table S1. HLA-DRB1 typing was performed by PCR sequence-specific primers (PCR-SSP) using custom oligonucleotides, with methods and primer sequences as described [70]. PCR products were photographed over ultraviolet light after electrophoretic separation on 1.5% agarose gels containing ethidium bromide. The method is based on the multiplexed amplification of both an allele-specific and a non-polymorphic sequence, used as internal control. Genotypes were deduced from the amplification patterns. HLA types were ascribed to a sample only when two alleles were amplified or, in the case of homozygosity, when internal controls were positive.

### Data analysis and statistics

Data analysis was performed on a Macintosh computer using the software IgorPro (WaveMetrics) and specially written procedures (Panama-Blot) for density quantification, migration scale adjustment, section definition and standardization of immunoblot data, as well as for statistics and coreferentiality calculations. Genotype scores for SNPs were defined as the number of minor alleles per individual (0, 1 or 2). For statistical testing, *P* values below 0.05 were considered significant. Group comparisons were performed by the distribution-independent Mann-Whitney or Kruskal-Wallis tests. Correlations were in principle described and tested for significance by distribution-independent Spearman rank correlation coefficients. Group-wise median correlations with immunoblot reactivities (Fig. 5), coreferentiality calculations and the according modeling, however, were based on linear (Pearson) correlations. Weighted correlations were used in order to adjust for familiar relatedness, i.e., to avoid representation bias of genetic or otherwise shared properties of related individuals, and to consider each family/pedigree with an equal weight of one. Weights were accordingly determined for each individual as the inverse of the total number of subjects in this individual's family/pedigree considered in the respective calculation. Within unrelated control subjects, no weighting was used since these subjects were all unrelated to each other.

Coreferentiality coefficients *R_C_* between pairs of test parameters were assessed and tested, resulting in a permutation *P*-value *P_Perm_*, as described [40], in respect to 130 quantified immunoblot reactivities as reference data. First-order linear correlation coefficients in these calculations were family-weighted, except within controls. For coreferentiality with all SNPs in a locus simultaneously, an analogous permutation test was performed that used the absolute maximal coreferentiality obtained among all considered SNPs as the test criterion instead of the absolute *R_C_*. Group-wise median correlations were also tested analogously, with the median *R* as test criterion. Bystander coreferentiality, i.e., the possibility that coreferentiality was generated by direct correlation between the test parameters with only one of them being related to the reference data [40], was checked for significant coreferentiality tests (except when for multiple SNPs) as follows. For each of the two test parameters, 100 artificial second parameter data sets were simulated, distributed N(0,10) and with family-weighted correlations with the first parameter being equal (±0.005) to those of the true respective second test parameter. Each simulation was then tested for coreferentiality, and the proportion of simulations reaching the significance level of the true data determined. In absence of a significant bystander effect, this proportion was expected to remain below 5%. This was always found to be the case.

### Modeling

Modeling was based on coreferentiality maximization either between a parameter and a function, or between two functions, each function representing the linear combination of several SNP genotype scores obtained from a respective locus. Functions had the form 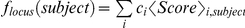, with *i* indicating the SNPs considered, and <Score> their respective genotype scores. Coefficients *c_i_* were varied in order to maximize coreferentiality, using the optimization algorithm ‘Amoeba’ adapted from [71]. For coreferentiality maximization, we first applied this algorithm 20 times using independent random initial estimates, and further 15 times on the basis of already optimized coefficients from the respective previous run as initial estimates, starting with those giving the highest obtained coreferentiality among the 20 first runs and stepwise tightening the convergence criterion. This procedure was tested and robustly yielded identical models when repeated with the same data and using up to 8 variable coefficients in the functions. SNPs included in the models were selected as follows. For the *IL2* locus, we had good evidence that variant alleles of *rs11575812*, *rs2069762*, and *rs2069763* excluded each other in the same haplotype (see discussion), and that their possible funcional effects were consequently independent. Accordingly, we modeled the *IL2* genetic effect as the linear combination of these three genotype scores. The effect of *IL2RA* genetic variation was modeled by selecting, from total 25 SNPs typed (Table S1), five (*rs11594656*, *rs10795791*, *rs7073236*, *rs706778*, *rs791589*) showing absolute coreferentialities above 0.5 with the dominant *IL2* SNP *rs11575812*, disconsidering a sixth SNP (*rs7072793*) that also met this criterion but was in almost complete LD with *rs10795791*. To obtain model scores for all subjects, including those not used in the model optimization, for secondary calculations, respective functions with the optimized model coefficients were applied to the individual genotype scores. For this purpose, some missing individual genotypes, not calling in Sequenom typing, were imputed using the software fastPhase™. In order to model *CD247* genetic effects for secondary model comparison, among a total of 24 SNPs typed, we selected three (*rs858535*, *rs1737506*, *rs863455*) that had no evident linkage between each other but covered the 5′ region of the locus where many single SNPs had high coreferentiality in the control group with the unaffected-relatives optimized *IL2RA* model score.

## Supporting Information

Figure S1 **Example of a quantitative immunoblot membrane.** Each membrane is incubated with up to 28 plasma samples, diluted to identical total protein concentrations of 1 mg/mL. In the shown picture after substrate development, band profiles of diverse individual samples can be seen. On each membrane, two channels as indicated were reserved for a unique reactivity standard serving to adjust resulting optical densities between membranes. One further channel was always used for an additional external quality control that was also present on a second membrane.
(PDF)

Figure S2 **Linkage disequilibrium map of the** ***IL2*** **locus according to our data (produced by HaploView). Annotations indicate pairwise R^2^ values.**
(PDF)

Figure S3 **Cytograms of confirmatory combined Foxp3/CD25 stainings of 15 individual SLE patients.** This figure is included to demonstrate that possibly activated and CD25-expressing Foxp3^−^conventional T-helper cells do not significantly contaminate the cells in our CD25^bright^ aTreg gate (R4) defined as described in the methods. Inserts in each panel indicate the respective proportions of Foxp3^+^ within CD25^bright^ cells. The uppest left cytogram, where Foxp3^−^CD25^+^ are most clearly present, shows that this presence does not involve a reduced proportion of Foxp3^+^ cells in the aTregs that we analyze.
(PDF)

Table S1 **Typed IL2, IL2RA and CD247 SNPs.**
(PDF)
