# Supplementary material for: Compensatory T-Cell Regulation in Unaffected Relatives of SLE Patients, and Opposite IL-2/CD25-Mediated Effects Suggested by Coreferentiality Modeling
Source: PLoS One. 2012 Mar 29;7(3):e33992. doi: 10.1371/journal.pone.0033992 (PMC3315511; doi:10.1371/journal.pone.0033992)

Figure S1. Example of a quantitative immunoblot membrane. Each membrane is incubated with up to 28 plasma samples, diluted to identical total protein concentrations of 1 mg/mL. In the shown picture after substrate development, band profiles of diverse individual samples can be seen. On each membrane, two channels as indicated were reserved for a unique reactivity standard serving to adjust resulting optical densities between membranes. One further channel was always used for an additional external quality control that was alsopresent on a second membrane.

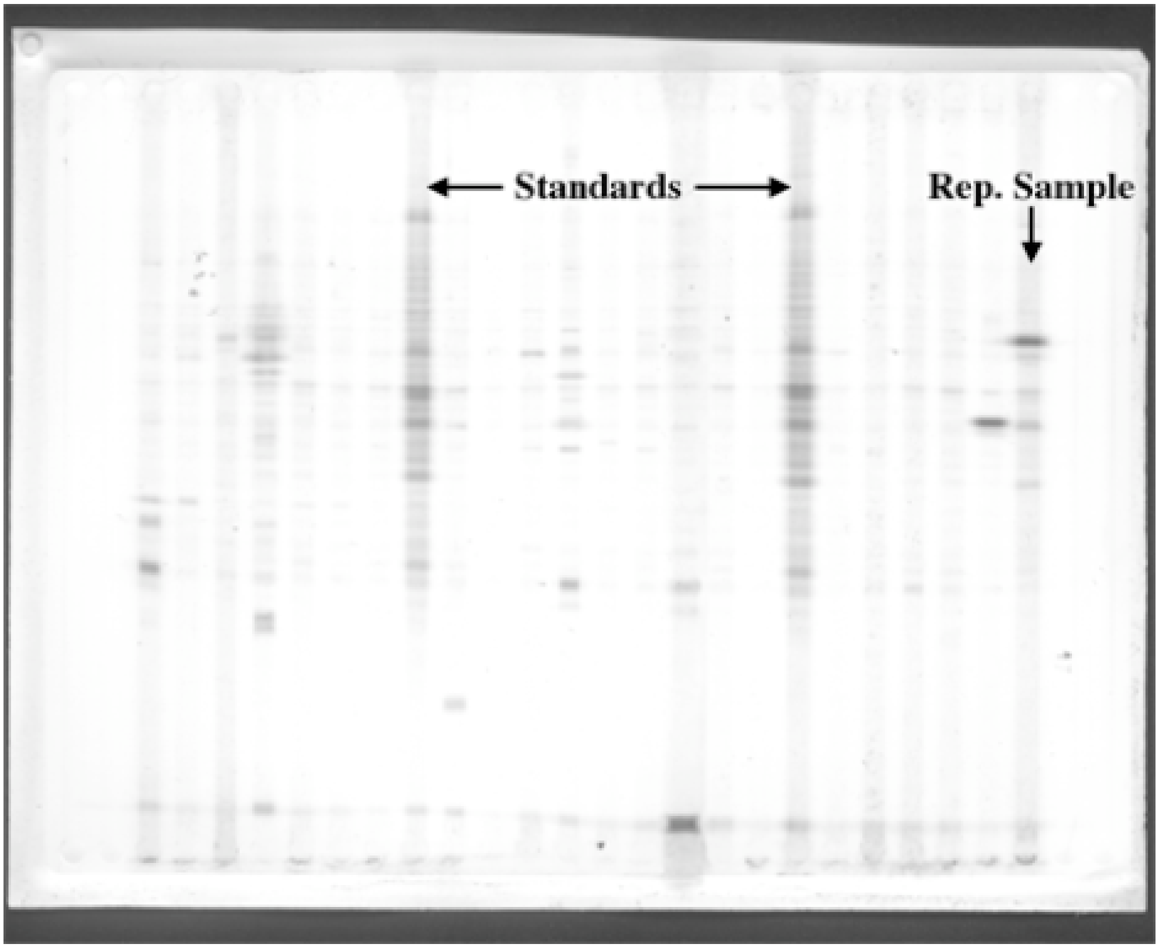

Supplement: Figure S1 — Example of a quantitative immunoblot membrane. Each membrane is incubated with up to 28 plasma samples, diluted to identical total protein concentrations of 1 mg/mL. In the shown picture after substrate development, band profiles of diverse individual samples can be seen. On each membrane, two channels as indicated were reserved for a unique reactivity standard serving to adjust resulting optical densities between membranes. One further channel was always used for an additional external quality control that was also present on a second membrane. (PDF) [file pone.0033992.s001.pdf]
