# Supplementary material for: Compensatory T-Cell Regulation in Unaffected Relatives of SLE Patients, and Opposite IL-2/CD25-Mediated Effects Suggested by Coreferentiality Modeling
Source: PLoS One. 2012 Mar 29;7(3):e33992. doi: 10.1371/journal.pone.0033992 (PMC3315511; doi:10.1371/journal.pone.0033992)

Figure S2. Linkage disequilibrium map of the *IL2* locus according to our data (produced by HaploView). Annotations indicate pairwise R^2 values.

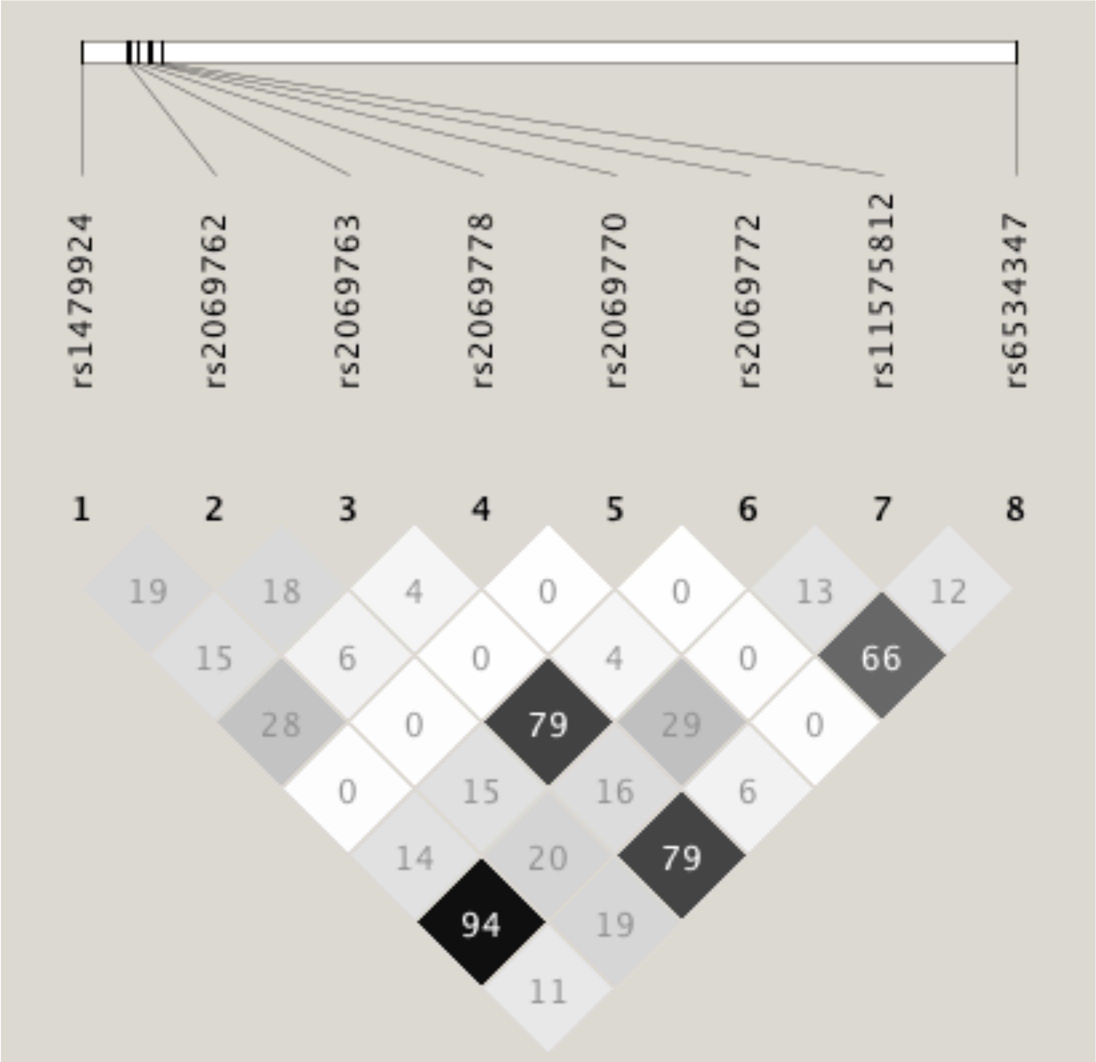

Supplement: Figure S2 — Linkage disequilibrium map of the IL2 locus according to our data (produced by HaploView). Annotations indicate pairwise R2 values. (PDF) [file pone.0033992.s002.pdf]
