# Supplementary material for: Compensatory T-Cell Regulation in Unaffected Relatives of SLE Patients, and Opposite IL-2/CD25-Mediated Effects Suggested by Coreferentiality Modeling
Source: PLoS One. 2012 Mar 29;7(3):e33992. doi: 10.1371/journal.pone.0033992 (PMC3315511; doi:10.1371/journal.pone.0033992)
Supplement: Figure S3 — Cytograms of confirmatory combined Foxp3/CD25 stainings of 15 individual SLE patients. This figure is included to demonstrate that possibly activated and CD25-expressing Foxp3−conventional T-helper cells do not significantly contaminate the cells in our CD25bright aTreg gate (R4) defined as described in the methods. Inserts in each panel indicate the respective proportions of Foxp3+ within CD25bright cells. The uppest left cytogram, where Foxp3−CD25+ are most clearly present, shows that this presence does not involve a reduced proportion of Foxp3+ cells in the aTregs that we analyze. (PDF) [file pone.0033992.s003.pdf]

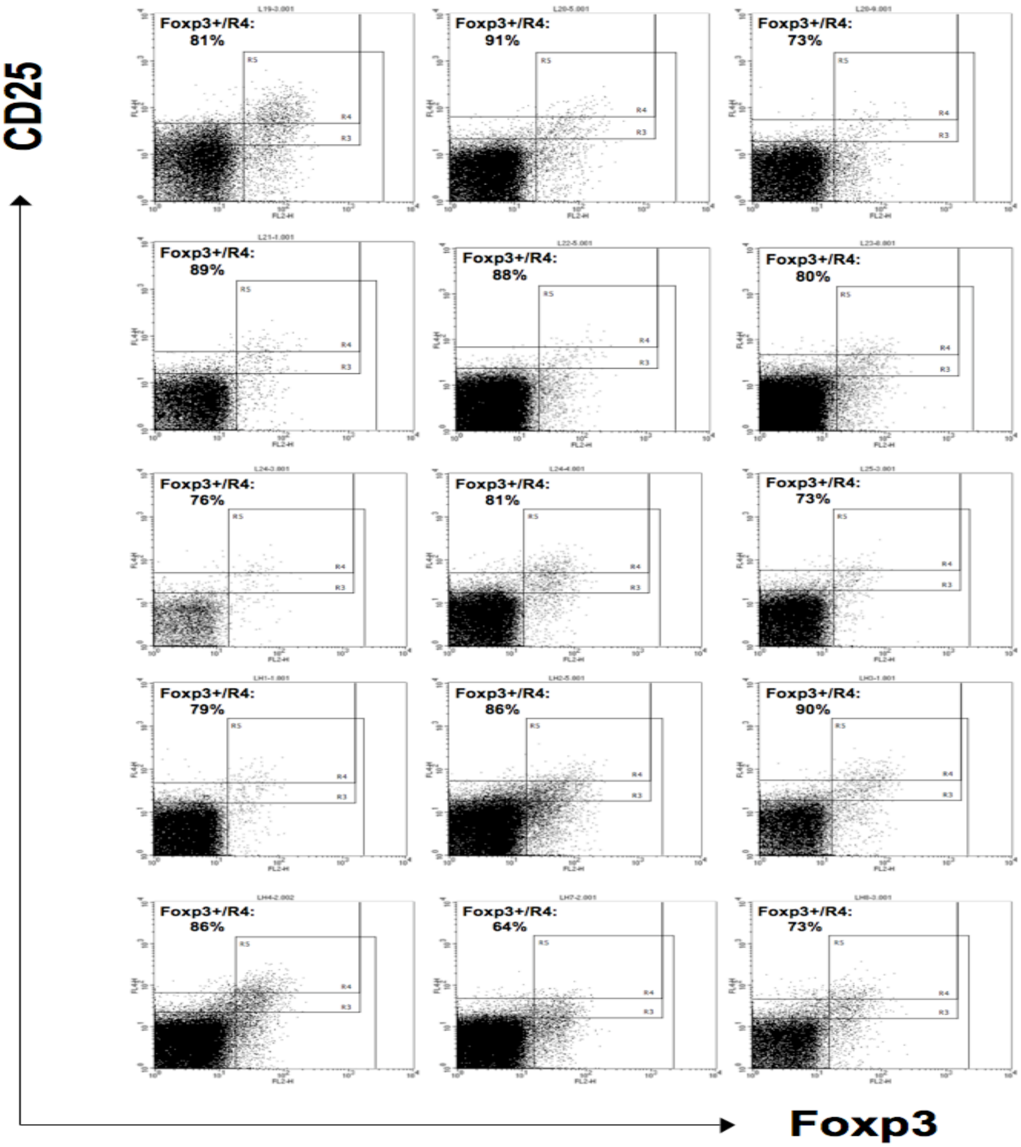

Fig. S3

Cytograms of confirmatory combined Foxp3/CD25 stainings of 15 individual SLE patients. This figure is included to demonstrate that possibly activated and CD25-expressing Foxp3^{-}conventional T-helper cells do not significantly contaminate the cells in our CD25^{bright} aTreg gate (R4) defined as described in the methods. Inserts in each panel indicate the respective proportions of Foxp3^{+} within CD25^{bright} cells. The uppest left cytogram, where Foxp3^{-}CD25^{+} are most clearly present, shows that this presence does not involve a reduced proportion of Foxp3^{+} cells in the aTregs that we analyze.
