# Supplementary material for: Compensatory T-Cell Regulation in Unaffected Relatives of SLE Patients, and Opposite IL-2/CD25-Mediated Effects Suggested by Coreferentiality Modeling
Source: PLoS One. 2012 Mar 29;7(3):e33992. doi: 10.1371/journal.pone.0033992 (PMC3315511; doi:10.1371/journal.pone.0033992)
Supplement: Table S1 — Typed IL2, IL2RA and CD247 SNPs. (PDF) [file pone.0033992.s004.pdf]

Table S1: Typed IL2, IL2RA and CD247 SNPs

Locus	SNP	Position	Coreferentiality with *rs11575812* in unaffected relatives	*IL2-IL2RA* Model coefficient	*CD247-IL2RA* Model coefficient	HWE* P-value (Controls)	Reported associations
IL2	rs1479924	123607050				1.0	
	**rs2069762**	**123597430**		**+0.03**		0.34	
	**rs2069763**	**123596932**		**-0.47**		0.06	SLE (ref. 39)
	rs2069778	123595585				1.0	
	rs2069770	123593197				1.0	
	rs2069772	123592583				0.19	
	**rs11575812**	**123590499**		**-0.88**		0.66	
	rs6534347	123417885				0.09	
IL2RA	rs12251307	6163501	-.20			0.04	
	**rs11594656**	**6162015**	**+.52**	**+0.14**	**+0.10**	0.29	**T1D, suspected causal (ref. 40), MS (ref. 59)**
	rs41295061	6154666	+.07			0.10	T1D, suspected causal (ref. 40), MS (ref. 59)
	rs7090530	6150881	-.29			0.81	
	rs4147359	6148445	-.21			0.72	
	**rs10795791**	**6148346**	**+.58**	**-0.77**	**-0.74**	0.85	
	rs11597367	6147540	-.35			0.65	
	**rs7073236**	**6146558**	**-.55**	**+0.51**	**+0.55**	0.70	
	rs7072793	6146272	-.53			0.98	
	rs2104286	6139051	+.30			0.15	RA and T1D (ref. 60), MS (ref. 59)
	**rs706778**	**6138955**	**+.60**	**+0.15**	**+0.21**	0.39	**T1D (ref. 60)**
	rs706779	6138830	+.15			0.41	
	rs2256774	6137171	+.25			0.41	
	rs10905669	6132099	+.13			0.79	
	**rs791589**	**6129577**	**+.56**	**-0.33**	**-0.31**	0.66	
	rs791587	6128705	-.15			0.20	
	rs11256497	6127800	+.31			0.27	
	rs706781	6126391	-.13			0.19	
	rs4749924	6122402	-.06			1.0	
	rs7072398	6119852	-.21			1.0	
	rs11256369	6106206	-.01			1.0	
	rs2076846	6103259	-.02			0.98	
	rs9663421	6095610	-.23			0.03	
	rs12244380	6093380	+.38			1.0	
	rs12359875	6091513	-.26			0.04	
CD3z	rs2143302	165758811				1.0	
	**rs858535**	**165758109**			**+0.71**	0.87	
	rs863454	165755423				0.89	
	rs2982481	165749930				0.40	
	rs1799704	165742495				0.92	
	**rs1737506**	**165740049**			**+0.03**	0.96	
	rs858543	165733922				0.32	
	rs10918706	165732745				0.54	
	rs704848	165728497				0.12	
	rs858545	165728015				0.50	
	**rs863455**	**165724448**			**+0.70**	0.22	
	rs858554	165721538				0.29	
	rs16859085	165719958				0.76	
	rs2949655	165718474				0.23	
	rs1214611	165715728				0.78	
	rs10918695	165704577				0.02	
	rs12036775	165703671				0.03	
	rs2995091	165700901				0.02	
	rs1723015	165699518				0.01	
	rs7523907	165693871				0.06	
	rs2056626	165687049				0.22	
	rs2995082	165672869				1.0	
	rs1917534	165663895				0.18	
	rs2949666	165659326				0.54	

*P-Value for Hardy-Weinberg equilibrium in control group
